# Abdominal aortic calcification on a plain X-ray and the relation with significant coronary artery disease in asymptomatic chronic dialysis patients

**DOI:** 10.1186/s12882-017-0480-2

**Published:** 2017-03-02

**Authors:** M. K. de Bie, M. S. Buiten, J. I. Rotmans, M. Hogenbirk, M. J. Schalij, T. J. Rabelink, J. W. Jukema

**Affiliations:** 1 Department of Cardiology, Leiden University Medical Center, PO Box 9600, 2300 RC Leiden, The Netherlands; 2 Department of Nephrology, Leiden University Medical Center, Leiden, The Netherlands; 3 Department of Nephrology, Rijnstate Ziekenhuis, Arnhem, The Netherlands

**Keywords:** Dialysis, Coronary Artery disease, Abominal aortic calcification, Vascular calcification

## Abstract

**Background:**

Coronary artery disease (CAD) is common in asymptomatic chronic dialysis patients and plays an important role in their poor survival. Early identification of these high-risk patients could improve treatment and reduce mortality. Abdominal aortic calcification (AAC) has previously been associated with CAD in autopsy studies. Since the AAC can be quantified easily using a lateral lumbar X-ray we hypothesized that the extent of AAC as assessed on a lateral lumbar X-ray might be predictive of the presence of significant CAD in dialysis patients.

**Methods:**

All patients currently enrolled in the ICD2 trial without a history of CABG or a PCI with stent implantation were included in this study. All patients underwent CT-angiography (CTA) and a lateral X-ray of the abdomen. AAC on X-ray was quantified using a previously validated scoring system whereupon the association between AAC and the presence of significant CAD was assessed.

**Results:**

A total of 90 patients were included in this study (71% male, 67 ± 7 years old). Forty-six patients were found to have significant CAD. AAC-score was significantly higher in patients with CAD (10.1 ± 4.9 vs 6.3 ± 4.6 (*p* < 0.05). Multivariate regression analysis revealed that AAC score is an independent predictor for the presence of CAD with a 1,2 fold higher risk per point increase (*p* < 0.01). The AAC score has a sensitivity of 85% and a specificity of 57% for the presence of significant CAD.

**Conclusion:**

This study shows that abdominal aortic calcification as assessed on a lateral lumbar X-ray is predictive for the presence of significant coronary artery disease in asymptomatic dialysis patients. This simple, non-invasive and cheap screening method could contribute to early identification of patients eligible for further screening of CAD.

**Trial registration:**

NTR948, registered 10-4-2007 ; ISRCTN20479861, registered 2-5-2007

## Background

Occlusive coronary artery disease (CAD) contributes significantly to the poor survival of chronic dialysis patients [1–3]. Optimizing treatment strategies for CAD could therefore substantially improve the outcome in this patient group. Although it is known that CAD is highly prevalent among dialysis patients, the current reported prevalence is probably an underestimation of the actual prevalence. Several studies have indicated that, also among asymptomatic dialysis patients, CAD is prevalent in approximately 40–50% of the patients [4–6]. Identification of these patients would allow for earlier and more optimal treatment. In dialysis patients it has been recently demonstrated that aortic calcification is an independent predictor of cardiovascular morbidity and mortality. It was therefore suggested that this screening modality could be used for accurate cardiovascular risk estimation in dialysis patients [7, 8]. Next to this relationship with cardiovascular events it has also been demonstrated, in an autopsy study performed in >600 middle aged adults, that the degree of abdominal calcification is associated with the extent of calcified coronary plaques [9].

Given these relationships, we hypothesized, that the extent of aortic calcification, as assessed on a plain lateral lumbar X-ray, might be predictive of the presence of significant CAD, in dialysis patients. The purpose of this study was to assess the predictive value of abdominal calcification for the presence of significant CAD using a validated scoring system, that has previously been used to assess the clinical value of abdominal aortic calcification [7].

## Methods

### Study population

For this analysis all patients enrolled in the ICD2 trial (ISRCTN20479861) until June 2013 were included. The background, objectives and methods of this study have been previously reported [10]. In summary, this study will evaluate the effectiveness of prophylactic ICD implantation in chronic dialysis patients. Before patients are randomized, an intensive screening protocol is performed, including computed tomography angiography (CTA) and a lateral lumbar X-ray. All patients provided written informed consent and the design of the trial was approved by the local ethics committee. Patients with a previous coronary artery bypass graft (CABG) or percutaneous coronary intervention (PCI) with stent implantation were not included, as were patients with an aortic prosthesis and patients in whom the CT-scan was not feasible, or considered uninterpretable.

#### Multi Slice CT protocol and MSCT data analysis

Prior to CT acquisition, patients with a high heart rate, defined as >65 beats per minute, received oral oral β-blockers (metoprolol 50 or 100 mg, single dose, 1 h before examination), if tolerated. Depending on the residual kidney function, pre and post procedural measures were taken in order to prevent further deterioration. These measures included pre and post procedural hydration (dose and route depending on the patients residual kidney function) and moreover in hemodialysis patients the scan was performed on the day prior to the next dialysis session.

Examinations were performed with a 64-detector row CT Scanner (Aquilion 64, Toshiba Medical Systems, Tokyo, Japan) or a 320-detector row CT scanner (Aquilion ONE, Toshiba, Tokyo, Japan) as previously described [11].

Data analysis was performed by two experienced CT observers (Including JWJ). If there was no consensus between these two reviewers a 3^rd^ independent reviewer was consulted. Data of all major epicardial segments (in the RCA segments 1–3; in the LAD segments 5–8; and in the LCx segments 11 and 13) was analysed as previously described. Significant CAD was defined as coronary luminal narrowing of ≥ 50% [11].

#### Quantification of abdominal aortic calcification

**Fig. 1:**
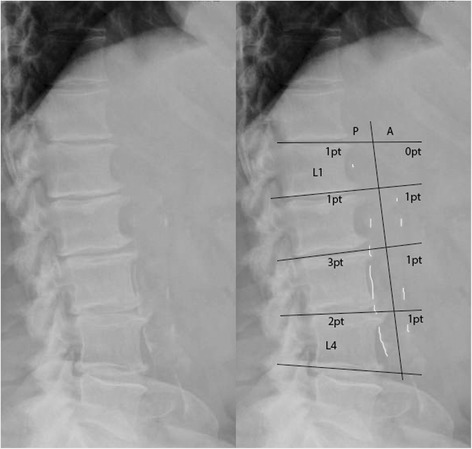
Grading of abdominal aortic calcification. For all segments both the anterior and posterior wall are graded for the extent of vascular calcification. Grading: 0 points: No calcific deposits; 1 point: less than 1/3 of the wall calcified; 2 points : between 1/3 and 2/3 of the wall affected; 3 points : more than 2/3 of the wall affected. The *right panel* shows an example of this calculation

The extent of aortic calcification was calculated on a lateral lumbar X-ray (MKB & MSB). The lateral X-ray was taken in a standing position using standard radiographic equipment. The grading was performed using a previously validated grading system [12, 13] in which the extent of calcific deposits is graded on a per segment basis using the lumbar vertebral segments L1-L4. Per segment a score between 0 and 3 was given for both the anterior and posterior wall of the Aorta. These eight scores resulted in a composite abdominal aortic calcification score (AAC score) ranging between 0 and 24 points (see Fig. 1).

#### Statistical analysis

Continuous variables are expressed as mean ± SD when normally distributed as assessed by Kolmogorv-Smirnov test. Non-normally distributed data were expressed as median (25^th^ and 75^th^ percentiles Q1,Q3). Continuous data were compared using the 2-tailed Student’s *t*-test for unpaired data or using the Mann–Whitney U-test when non-normally distributed. Categorical data were expressed as frequencies and percentages and were compared using the Chi-square test. Using logistic regression analysis, the univariate predictive value of the baseline parameters for the presence of coronary artery disease, was assessed. Subsequently a multivariate analysis (controlling for all univariate predictors with *p* < 0.2) was performed. All statistical analyses were performed using SPSS (version 20.0, SPPS Inc. Chicago, Illinois). All statistical tests were two-sided and a *p*-value <0.05 was considered statistically significant.

## Results

For this study 90 out of the 142 patients enrolled in the ICD2 study were eligible for this analysis; 23 patients were not included because of a history of CABG, 10 patients because of a history of PCI with stent implantation, six patients because of a high/irregular heart rate, four patients had an non-diagnostic CTA, three patients had an aortic prosthesis and four patients refused CTA. Furthermore in two patients no lateral lumbar X-ray was obtained.

Patients were predominantly male (71%) with an average age of 67 ± 7 years. Most patients were on haemodialysis (69%) for a median duration for 18 [9, 29] months. The average abdominal aortic calcification score was 8.2 ± 5.1 points (range 0–20.5 points). The baseline characteristics are summarized in Table 1. No significant adverse events relating to the CT angiography or lumbar X-ray were documented.

**Table 1:** Baseline characteristics

	All (*n* = 90)	No CAD (*n* = 44)	CAD (*n* = 46)
Age, yrs	67 ± 7	65 ± 7	69 ± 7*
Male gender, nr.(%)	64 (71%)	27 (61%)	37 (80%)
Hemodialysis, nr.(%)	62 (69%)	29 (66%)	33 (72%)
Dialysis Vintage, months	18 [9, 29]	17 [9, 33]	18 [8, 25]
BMI	26.8 ± 4.4	26.8 ± 4.5	26.8 ± 4.3
Hypertension, nr (%)	70 (77%)	32 (73%)	38 (83%)
Diabetes, nr (%)	26 (29%)	12 (27%)	14 (30%)
History of smoking, nr (%)	60 (67%)	28 (64%)	32 (70%)
Beta-Blocker, nr (%)	45 (50%)	23 (52%)	22 (48%)
ACEi/ARB, nr (%)	48 (53%)	21 (48%)	27 (59%)
Statin, nr (%)	45 (50%)	21 (48%)	24 (52%)
Troponin I (ng/L)	12 [7–22]	12 [6–24]	13 [7–17]
Troponin T (ng/L)	48 [32–75]	42 [29–71]	55 [35–81]
LV Ejection Fraction (%)	54% ± 6%	54% ± 7%	53% ± 6%
LVMi (g/m2)	125 ± 40	128 ± 45	122 ± 34
CACS	690 [133, 2085]	279 [20, 1691]	912 [441, 2217]*
AAC-score	8.2 ± 5.1	6.3 ± 4.6	10.1 ± 4.9*

*CAD* coronary artery disease, *BMI* body mass index, *ACEi* angiotensin converting enzyme inhibitor, *ARB* angiotensin receptor blocker, *LV* left ventricular; *LVMi* left ventricular mass indexed for body surface area, *CACS* coronary artery calcification score, *AAC* abdominal aortic calcification; * = *p* <0.05

### Coronary artery disease

Significant coronary artery disease was documented in 46 (51%) of the patients. Compared to patients with no significant CAD, these patients were significantly older (69 ± 7 vs. 65 ± 7 years, *p* <0.05). Furthermore patients with CAD were predominantly male (80 vs. 61%, *p* < 0.05). The AAC score was significantly higher in patients with significant CAD measured by CTA (10.1 ± 4.9 vs 6.3 ± 4.6 points, *p* <0.05). As could be expected the coronary artery calcium score assessed by CT was also higher in patients with CAD.

### Prediction of the presence of coronary artery disease

Using logistic regression analysis, the univariate predictive value of the baseline parameters, for the presence of CAD, was assessed. Older age, male gender and AAC score were predictors for the presence of significant CAD. Multivariate analysis demonstrated that the AAC score was a significant and independent predictor for the presence of significant CAD with an approximately1.2 fold higher risk per point increase (Table 2). Of interest, also a significant correlation was found between the AAC score and the Coronary Calcium score. (Correlation Coefficient 0.45, *p* < 0.01, Spearman’s rho).

**Table 2:** Uni- and multivariate predictors for the presence of CAD

	Univariate	Multivariate
Age	1.06 (1.00–1.13), *p* < 0.05	1.02 (0.95–1.09), *p* = 0.64
Male gender	2.59 (1.00–6.68), *p* < 0.05	2.73 (0.95–7.82), *p* = 0.062
Hemodialysis	1.31 (0.54–3.20), *p* = 0.55	
Dialysis vintage	1.0 (0.99–1.00), *p* = 0.42	
AACscore (per point)	1.19 (1.07–1.30), *p* < 0.05	1.18 (1.06–1.32), *p* < 0.01
Diabetes	1.17 (0.47–2.90), *p* = 0.74	
History of smoking	1.31 (0.54–3.14), *p* = 0.55	
Hypertension	1.78 (0.65–4.89), *p* = 0.26	
BMI	1.0 (0.91–1.10), *p* = 0.95	

*AAC* abdominal aortic calcification, *BMI* body mass index

### Sensitivity and Specificity

**Fig. 2:**
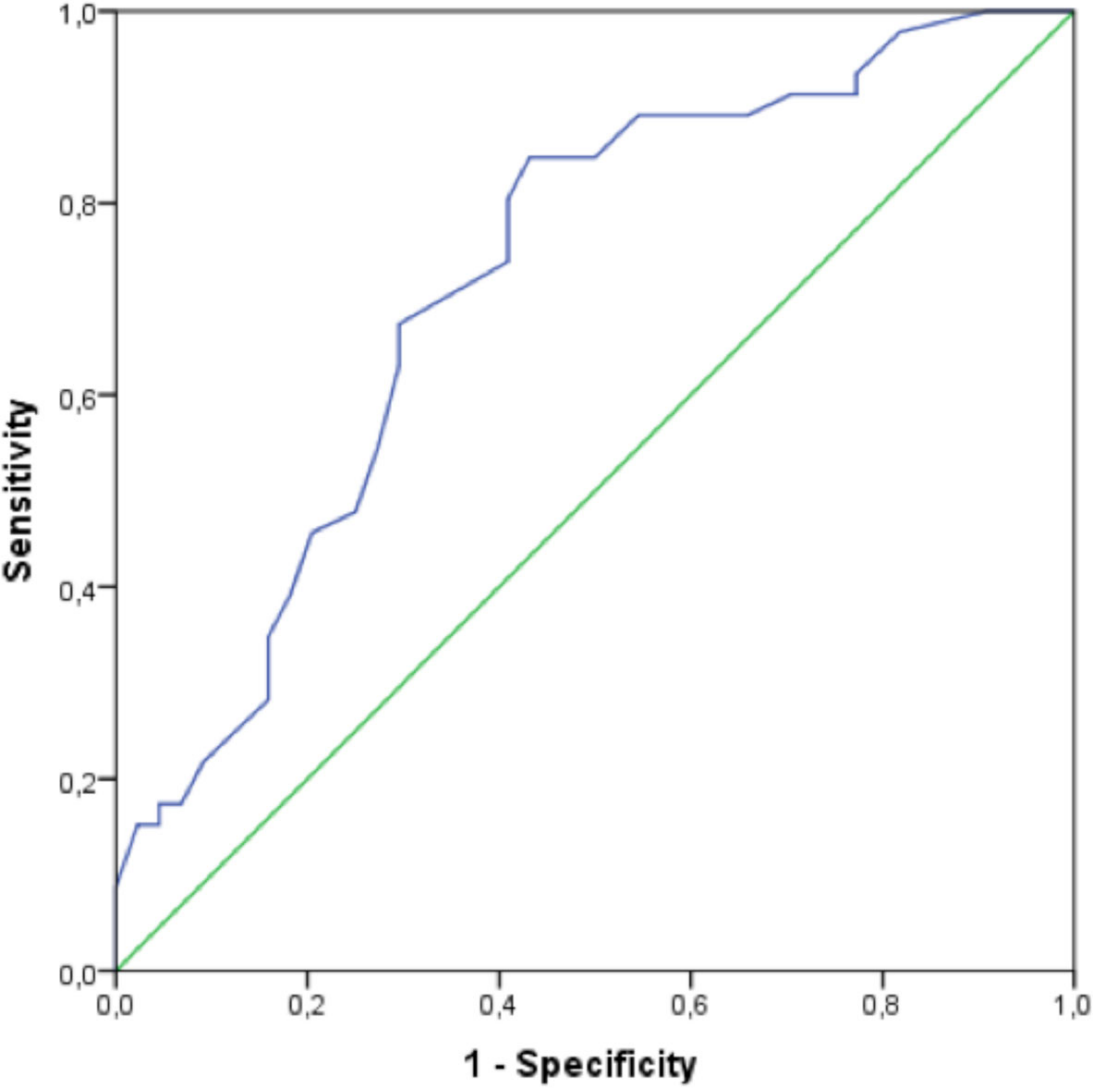
ROC-analysis of AAC-score

Using ROC curve analysis, the optimal cut-off for the AAC score was assessed. With a cut-off of 6.5 points, the AAC score had a sensitivity of 85% and a specificity of 57% for predicting the presence of significant CAD. Furthermore when this cut-off is used, the AAC score has a negative predictive value of 78% and a positive predictive value of 67% (Table 3). Receiver operating characteristics curve analysis demonstrated an area under the curve of 0.72 (*p* <0.05). See Fig. 2.

**Table 3:** Presence of significant CAD using an AACscore cutoff of 6.5 points

	CAD	No CAD	
AACscore ≥6.5	39	19	*PPV :* 67%
AACscore <6.5	7	25	*NPV :* 78%
	*Sensitivity* : 85%	*Specificity* : 57%	

*CAD* coronary artery disease, *AACscore* abdominal aortic calcification score

When this cut-off was entered in the multivariable model, as described previously, an AAC score ≥ 6.5 was associated with a 6.56 (95% CI 2.28–18.85, *p* < 0.001) fold increased risk for the presence of significant CAD.

## Discussion

This study demonstrates that the extent of abdominal aortic calcification, assessed using a plain lateral abdominal X-ray, is predictive for the presence of significant CAD in chronic dialysis patients.

### The necessity to detect significant CAD in dialysis patients

CAD is highly prevalent among dialysis patients and is a major contributor to the poor outcome of dialysis patients [2, 14]. Optimal treatment of CAD could however, lead to significant improvements. For instance it has been demonstrated that revascularization in dialysis patients in whom significant CAD is detected may indeed increase survival [15–17]. Nevertheless, despite these observations, it should be noted that it has been reported that invasive treatment for CAD is underused in dialysis patients [18].

Since significant CAD is highly prevalent among asymptomatic dialysis patients a substantial part is probably not optimally treated. It is possible that these patients would benefit from more intensive medical treatment or even revascularization. Some controversy remains on this topic however, since the definition of optimal treatment in this patient group is not a clear cut case. Further studies are needed in the future to address this problem. Although it has not been demonstrated, it seems reasonable to believe that, given the high incidence of CAD among dialysis patients and the fact that some patients probably would benefit of more intensive treatment, screening for CAD is warranted in dialysis patient in order to optimize treatment for CAD. Nonetheless, the true value of screening for CAD in dialysis patients needs to be addressed in future research.

### Abdominal aortic calcification and coronary artery disease

Already in the 1950s it was reported, based on autopsy studies of > 600 middle aged adults, that there is a highly significant association between the degree of abdominal aortic calcification and the presence of calcified plaque in the coronary arteries [9]. Following these findings it was demonstrated, in the general population, that using a plain lateral abdominal x-ray the severity of aortic calcification can be predicted. Moreover a relationship between the extent of aortic calcification found on lateral lumbar x-ray and cardiovascular morbidity and mortality was reported [13, 19]. Consequently this relationship was also documented in dialysis patients [7]. To our knowledge this is the first study to demonstrate that the AAC score is predictive for the presence of significant CAD in dialysis patients.

### Other modalities to detect significant CAD

Many modalities to detect CAD are currently available, however each of them has its own limitations. The gold standard for the diagnosis of CAD, coronary angiography (CAG), has been suggested as a routine screening tool of CAD in dialysis patients [5, 20]. However, given its invasive nature, the high costs and the risks of complications, other diagnostic tools would probably be preferable in this asymptomatic population. Other imaging modalities have been proposed as well, such as dobutamine stress echocardiography and myocardial perfusion scintigraphy [21].

Although no true comparison can be made between the screening modality presently investigated and other diagnostic tools for coronary artery disease, it is presumable that a plain lateral lumbar X-ray has financial and logistical advantages over the other diagnostic screening modalities mentioned.

### Clinical implications

CAD is highly prevalent among dialysis patients but it has not yet been decided which diagnostic modality is preferable. However, routine screening for CAD, using one of the many available screening modalities seems justified, in order to improve the abysmal outcome of this patient group. We showed that aortic calcification on plain lateral lumbar x-ray is predictive for the presence of significant CAD. Using this inexpensive, non-invasive screening method, patients with high risk of having significant CAD can be easily identified. Subsequently the most optimal diagnostic or treatment strategy could then be initiated. It should however, once more, be stated that future research should further investigate what these treatment strategies for CAD in dialysis patients should be, since this is not yet a clear cut case.

### Limitations

CT-angiography was used to detect the presence of significant CAD instead of coronary angiograpy (CAG). With CTA it is known that heavily calcified segments might give false positive results [22, 23]. On the other hand, in contrast to patients with normal renal function, in whom calcification occurs in the intima of the vessel, in dialysis patients calcification is often related to the media of the vessel [24, 25]. Since media calcification does not result in luminal narrowing, the luminal evaluation might still be feasible in dialysis patients. Moreover, it was also demonstrated that sensitivity and specificity of novel CT systems remain high despite severe coronary calcification. The authors of this meta-analysis suggest that diffuse calcifications resulting in a high calcium score are less likely to result in non-interpretability compared to considerable calcification in a small area [26]. In dialysis patients however, vascular calcification is usually a generalized problem. When analysing the feasibility of coronary CT-angiography we did not find a significant difference in coronary calcium score between patients with a completely interpretable scan and patients in whom one or more segments where considered non-interpretable [11]. In the current study 4 CT-scans where considered non-diagnostic, those patients where excluded from the current analysis.

Although it has been recently reported by our group that CT-angiography is feasible to detect CAD in dialysis patients, coronary angiograms still should be considered the gold standard [11]. Future studies therefore should correlate the AAC score to CAD detected by CAG in order to confirm our observations.

## Conclusion

Aortic calcification as assessed on a plain lateral lumbar X-ray is predictive for the presence of significant CAD in asymptomatic chronic dialysis patients. Using this X-ray, patients with high risk for CAD can be identified.

## Abbreviations

AAC: Abdominal aortic calcification
CABG: Coronary artery bypass grafting
CAD: Coronary artery disease
CAG: Coronary angiogrpahy
CTA: Computed tomography angiography
PCI: Percutaneous coronary intervention

## References

[1] U.S. Renal Data System. USRDS 2006 Annual Data Report: Atlas of Chronic Kidney Disease and End-Stage Renal Disease in the United States. Bethesda: National Institutes of Health, National Insitute of Diabetes and Digestive and Kidney Diseases; 2006

[2] Cheung AK, Sarnak MJ, Yan G, Berkoben M, Heyka R, Kaufman A (2004). Cardiac diseases in maintenance hemodialysis patients: results of the HEMO Study. Kidney Int.

[3] de Bie MK, van Dam B, Gaasbeek A, van Buren M, van Erven L, Bax JJ (2009). The current status of interventions aiming at reducing sudden cardiac death in dialysis patients. Eur Heart J.

[4] Charytan D, Kuntz RE, Mauri L, DeFilippi C (2007). Distribution of coronary artery disease and relation to mortality in asymptomatic hemodialysis patients. Am J Kidney Dis.

[5] Joki N, Hase H, Nakamura R, Yamaguchi T (1997). Onset of coronary artery disease prior to initiation of haemodialysis in patients with end-stage renal disease. Nephrol Dial Transplant.

[6] Ohtake T, Kobayashi S, Moriya H, Negishi K, Okamoto K, Maesato K (2005). High prevalence of occult coronary artery stenosis in patients with chronic kidney disease at the initiation of renal replacement therapy: an angiographic examination. J Am Soc Nephrol.

[7] Verbeke F, Van BW, Honkanen E, Wikstrom B, Jensen PB, Krzesinski JM (2011). Prognostic value of aortic stiffness and calcification for cardiovascular events and mortality in dialysis patients: outcome of the calcification outcome in renal disease (CORD) study. Clin J Am Soc Nephrol.

[8] Okuno S, Ishimura E, Kitatani K, Fujino Y, Kohno K, Maeno Y (2007). Presence of abdominal aortic calcification is significantly associated with all-cause and cardiovascular mortality in maintenance hemodialysis patients. Am J Kidney Dis.

[9] Eggen DA, Strong JP, McGill HC (1964). Calcification in the abdominal aorta; relationship to race, sex, and coronary atherosclerosis. Arch Pathol.

[10] de Bie MK, Lekkerkerker JC, van Dam B, Gaasbeek A, van Buren M, Putter H (2008). Prevention of sudden cardiac death: rationale and design of the Implantable Cardioverter Defibrillators in Dialysis patients (ICD2) Trial--a prospective pilot study. Curr Med Res Opin.

[11] de Bie MK, Buiten MS, Gaasbeek A, Boogers MJ, Roos CJ, Schuijf JD (2013). CT coronary angiography is feasible for the assessment of coronary artery disease in chronic dialysis patients, despite high average calcium scores. PLoS One.

[12] Kauppila LI, Polak JF, Cupples LA, Hannan MT, Kiel DP, Wilson PW (1997). New indices to classify location, severity and progression of calcific lesions in the abdominal aorta: a 25-year follow-up study. Atherosclerosis.

[13] Wilson PW, Kauppila LI, O'Donnell CJ, Kiel DP, Hannan M, Polak JM (2001). Abdominal aortic calcific deposits are an important predictor of vascular morbidity and mortality. Circulation.

[14] Foley RN, Parfrey PS, Sarnak MJ (1998). Clinical epidemiology of cardiovascular disease in chronic renal disease. Am J Kidney Dis.

[15] Hemmelgarn BR, Southern D, Culleton BF, Mitchell LB, Knudtson ML, Ghali WA (2004). Survival after coronary revascularization among patients with kidney disease. Circulation.

[16] Yasuda K, Kasuga H, Aoyama T, Takahashi H, Toriyama T, Kawade Y (2006). Comparison of percutaneous coronary intervention with medication in the treatment of coronary artery disease in hemodialysis patients. J Am Soc Nephrol.

[17] Kumar N, Baker CS, Chan K, Duncan N, Malik I, Frankel A (2011). Cardiac survival after pre-emptive coronary angiography in transplant patients and those awaiting transplantation. Clin J Am Soc Nephrol.

[18] Charytan D, Mauri L, Agarwal A, Servoss S, Scirica B, Kuntz RE (2006). The use of invasive cardiac procedures after acute myocardial infarction in long-term dialysis patients. Am Heart J.

[19] Witteman JC, Kok FJ, van Saase JL, Valkenburg HA (1986). Aortic calcification as a predictor of cardiovascular mortality. Lancet.

[20] Joki N, Hase H, Takahashi Y, Ishikawa H, Nakamura R, Imamura Y (2003). Angiographical severity of coronary atherosclerosis predicts death in the first year of hemodialysis. Int Urol Nephrol.

[21] De Vriese AS, Vandecasteele SJ, Van den Bergh B, De Geeter FW (2012). Should we screen for coronary artery disease in asymptomatic chronic dialysis patients?. Kidney Int..

[22] Ghostine S, Caussin C, Daoud B, Habis M, Perrier E, Pesenti-Rossi D (2006). Non-invasive detection of coronary artery disease in patients with left bundle branch block using 64-slice computed tomography. J Am Coll Cardiol.

[23] Raff GL, Gallagher MJ, O’Neill WW, Goldstein JA (2005). Diagnostic accuracy of noninvasive coronary angiography using 64-slice spiral computed tomography. J Am Coll Cardiol.

[24] Cannata-Andia JB, Rodriguez-Garcia M, Carrillo-Lopez N, Naves-Diaz M, Diaz-Lopez B (2006). Vascular calcifications: pathogenesis, management, and impact on clinical outcomes. J Am Soc Nephrol.

[25] Goldsmith D, Ritz E, Covic A (2004). Vascular calcification: a stiff challenge for the nephrologist: does preventing bone disease cause arterial disease?. Kidney Int..

[26] den Dekker MA, de Smet K, de Bock GH, Tio RA, Oudkerk M, Vliegenthart R (2012). Diagnostic performance of coronary CT angiography for stenosis detection according to calcium score: systematic review and meta-analysis. Eur. Radiol..

